# *Fmr1* exon 14 skipping in late embryonic development of the rat forebrain

**DOI:** 10.1186/s12868-022-00711-1

**Published:** 2022-05-31

**Authors:** Juliana C. Corrêa-Velloso, Alessandra M. Linardi, Talita Glaser, Fernando J. Velloso, Maria P. Rivas, Renata E P. Leite, Lea T. Grinberg, Henning Ulrich, Michael R. Akins, Silvana Chiavegatto, Luciana A. Haddad

**Affiliations:** 1 Department of Genetics and Evolutionary Biology, Instituto de Biociências, Universidade de São Paulo, Rua do Matão, 277 # 327, São Paulo, SP 05508-090 Brazil; 2 Department of Biochemistry, Instituto de Química, Universidade de São Paulo, São Paulo, SP Brazil; 3 Department of Pathology, Faculdade de Medicina, Universidade de São Paulo, São Paulo, SP Brazil; 4 Department of Biology, Drexel University, Philadelphia, PA USA; 5 Department of Pharmacology, Instituto de Ciências Biomédicas, Universidade de São Paulo, São Paulo, SP Brazil; 6 Department of Psychiatry, Instituto de Psiquiatria, Faculdade de Medicina, Universidade de São Paulo, São Paulo, SP Brazil

**Keywords:** Fmr1, Fragile X syndrome-alternative splicing, Nervous system, Forebrain, Cerebellum, Nonsense-mediated decay (NMD), UPF1

## Abstract

**Background:**

Fragile X syndrome, the major cause of inherited intellectual disability among men, is due to deficiency of the synaptic functional regulator FMR1 protein (FMRP), encoded by the FMRP translational regulator 1 (*FMR1*) gene. *FMR1* alternative splicing produces distinct transcripts that may consequently impact FMRP functional roles. In transcripts without exon 14 the translational reading frame is shifted. For deepening current knowledge of the differential expression of *Fmr1* exon 14 along the rat nervous system development, we conducted a descriptive study employing quantitative RT-PCR and BLAST of RNA-Seq datasets.

**Results:**

We observed in the rat forebrain progressive decline of total *Fmr1* mRNA from E11 to P112 albeit an elevation on P3; and exon-14 skipping in E17–E20 with downregulation of the resulting mRNA. We tested if the reduced detection of messages without exon 14 could be explained by nonsense-mediated mRNA decay (NMD) vulnerability, but knocking down UPF1, a major component of this pathway, did not increase their quantities. Conversely, it significantly decreased *FMR1* mRNA having exon 13 joined with either exon 14 or exon 15 site A.

**Conclusions:**

The forebrain in the third embryonic week of the rat development is a period with significant skipping of *Fmr1* exon 14. This alternative splicing event chronologically precedes a reduction of total *Fmr1* mRNA, suggesting that it may be part of combinatorial mechanisms downregulating the gene’s expression in the late embryonic period. The decay of *FMR1* mRNA without exon 14 should be mediated by a pathway different from NMD. Finally, we provide evidence of *FMR1* mRNA stabilization by UPF1, likely depending on FMRP.

**Supplementary Information:**

The online version contains supplementary material available at 10.1186/s12868-022-00711-1.

## Background

**Fig. 1:**
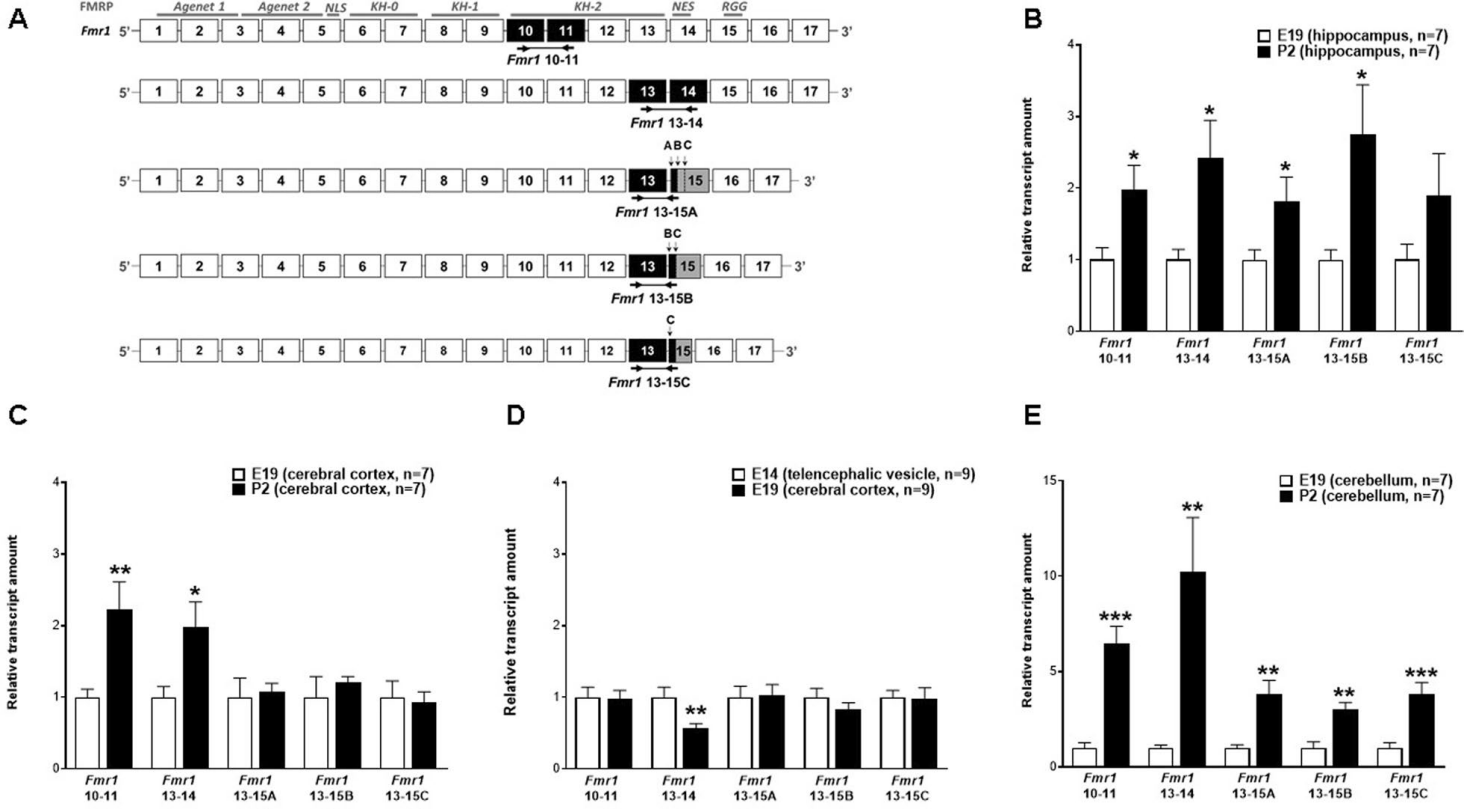
Expression of* Fmr1* exon 14 in the rat brain on E14, E19 or P2. **A** Full-length rat *Fmr1* exon organization and correspondence to encoded FMRP domains: two N-terminal Agenet domains, three central hnRNP K-homology (KH) domains (KH-0, KH-1 and KH-2), and a C-terminal region RGG-box. NLS and NES correspond to the nuclear localization and export signal motifs, respectively. Arrows indicate RTqPCR primers location on exons, defining amplicons (black boxes) for exons 10 and 11, and messages specifically containing *Fmr1* exon 14 or junctions between exons 13 and 15 by using the first (**A**), second (**B**), or third (**C**) splicing acceptor sites. **B–E** RTqPCR results of relative mRNA expression rates of the hippocampus (**B**), cerebral cortex (**C**), E14 telencephalic vesicle and E19 cerebral cortex (**D**), and cerebellum (**E**). Student´s *t* test: *P* < 0.05 (*), *P* < 0.01 (**), and *P* < 0.001(***)

Fragile X syndrome (OMIM #300624), the major cause of inherited intellectual disability among men, is due to deficiency of the synaptic function regulator FMR1 protein (FMRP; UniProt Q06787), encoded by the FMRP translational regulator 1 (*FMR1*, OMIM #*309550) gene. The most common mutation causing fragile X syndrome is the expansion of the trinucleotide CGG repeats in *FMR1* first exon into full mutation (more than 200 repeats) leading to transcription repression (reviewed by [1, 2]). In the central nervous system (CNS), FMRP is expressed mainly in the hippocampus, cerebral cortex and cerebellum [3, 4]. Accumulating evidence implicates FMRP in regulatory roles in neurogenesis, synapse development, elimination, and plasticity [5–11]. FMRP is an RNA-binding protein, functionally involved in many aspects of RNA homeostasis and notably in the mRNA translation control [12, 13]. These functions are mediated by different motifs and domains permitting the direct binding of RNA to FMRP (Fig. 1A) [1, 2, 14].

In the postnatal CNS, FMRP has been mostly described as a translational repressor of dendritic mRNAs in glutamatergic synapses, regulating synaptic plasticity, in particular long-term depression [8]. However, FMRP is observed in additional neuronal types [15, 16], distinct subcellular distribution such as in the axonal compartment [11, 13, 17], and other cell types as glia [18]. During cerebral corticogenesis of the mouse embryo, loss of Fmrp depletes radial glia cells by increasing their differentiation into intermediate progenitor cells [19], and affects the multipolar to bipolar neuronal transition in the cortical plate [20]. Therefore, the neurological deficits observed in fragile X syndrome patients can be due not only to synaptogenesis deficiency but also to neuronal differentiation abnormalities in earlier phases of the embryonic development.

The *FMR1* gene harbors 17 exons and maps to Xq27.3 [21]. The *FMR1* primary transcript may undergo alternative splicing by skipping exons 12 or 14, or selecting among three splicing acceptor sites in intron 14/exon 15 boundaries (15 A, 15B, 15 C; Fig. 1 A), and two splice acceptor sites in intron 16/exon 17 (17 A and 17B) [21–23]. Moreover, a recent study described that a deeply internal short segment of intron 9 can be maintained in human *FMR1* mature mRNA as a cryptic exon (exon 9a), specifically in leukocytes, which express a truncated isoform of FMRP [24].

*FMR1* exons 11 and 12 code for the variable loop of FMRP KH-2 domain (Fig. 1A). While *FMR1* exon 11 encodes a short form of the KH-2 domain variable loop, the alternative, in-frame expression of exon 12 produces a long form of the loop that has reduced RNA association [25, 26]. The highest mRNA levels of the endogenous full-length *FMR1*, expressing exon 12, have been observed in the mouse brain on E9 and in cultured neural progenitor cells [27]. Another study has seen increased expression of *FMR1* exon 12 in progenitor cells treated for neuronal differentiation [26], although the functional roles of exon-12-expressing isoforms have not been clarified. On the other hand, alternative selection among the three acceptor sites to splice exon 15 5’ end has been shown to effectively alter posttranslational modifications of FMRP. Use of site 15B, in comparison to 15 A, results in the absence of a key regulatory serine whose phosphorylation impacts FMRP association with ribosomes [28]. Use of site 15 C additionally results in lack of a methylation signal that is required for RNA binding through the exon 15-encoded RGG box [29].

Upon skipping of *FMR1* exon 14, exon 13 joins with any of the three exon 15 splice acceptor sites, shifting the translational reading frame [22]. Thus, *FMR1* mRNA exon 13 joining to exon 15 splicing acceptor sites 15A or 15B creates premature translational termination codons (PTC) on exon 15 [21], whereas with site 15C also shifts the reading frame, while producing PTC on exon 17 (Fig. 3A). It remains to be demonstrated if mature *FMR1* mRNA lacking exon 14 is more prone to RNA decay mechanisms (nonsense-mediated mRNA decay, NMD) as mediated by PTC [30]. As exon 14 codes for a nuclear export sequence (NES, Fig. 1A), its skipping should potentially alter FMRP subcellular localization [31, 32]. *FMR1* exon 15 encodes RNA-binding arginine-glycine-glycine (RGG) motifs [33] and key regulatory phosphorylation and methylation motifs [28, 29]. Therefore, *FMR1* exon 14 skipping shifting the reading frame might produce FMRP isoforms with variable C-termini without regulatory sites or RGG motifs, which should consequently have changes in responses to upstream regulators as well as to interactions with RNA, thus impacting downstream signaling. Although overexpressed human putative FMRP isoforms with novel C-termini have been detected in nuclear Cajal bodies [34], their potential endogenous nuclear roles remain unknown. As exon 14 skipping has been considered a rare alternative splicing event [27], it is not yet clear if skipping exon 14 in *FMR1* primary transcripts is a stochastic or developmentally regulated event.

*FMR1* alternative transcripts can potentially result in 20 non-redundant FMRP isoforms, named isoforms 1 to 20, according to  Sittler et al. [22]. However, working with endogenous FMRP isoforms is challenging as different protein bands are detected on Western blots by anti-FMRP antibodies and various posttranslational modifications have been described altering their migration in gel [28, 29]. Therefore, studies aiming at assessing the endogenous expression levels of *Fmr1* alternative exons have been based on mRNA quantification [26, 27].

In *Rattus norvegicus*, as in mouse, the *Fmr1* gene knockout impairs synaptic plasticity [6–9, 35, 36]. Twelve *Fmr1* variable transcripts have been individually assessed in the mouse brain by quantitatively characterizing the transcript output expected for each unique combination among the four alternative splicing events [27]. However, it is still unknown if there is a developmental period when skipping exon 14 is most significant. To analyze that, it is important to quantify the specific exon in *Fmr1* mRNA during definite developmental transition periods when FMRP function has been notably demonstrated, such as in telencephalic neurogenesis and in critical periods of synaptogenesis [11, 19, 37, 38]. Identifying a narrow developmental window in which this splicing event is significant can be informative to direct efforts to understand the functional roles of the variable transcripts. Furthermore, a broader developmental assessment of *Fmr1* mRNA is lacking. Here, we present a descriptive study on exon 14 expression rates in *Fmr1* mRNA by RT-qPCR in the rat brain, and the effect of knocking down NMD on messages lacking this exon. Moreover, we analyzed *in silico* rat RNA-Seq datasets for the relative quantity of total *Fmr1* mRNA and the alternative exon 14 and exon 15 splice sites. Altogether, our results in rat forebrain describe in the third embryonic week a significant reduction of *Fmr1* mRNA as well as exon 14. In addition, RNA-Seq data assessment provided an extensive quantitative mRNA profile of *Fmr1* in the rat CNS, displaying high amounts of the ensemble of its transcripts in early embryonic ages of whole brain that successively decreased through the postnatal period of the forebrain, apart from augmentation on postnatal (P) day P3.

## Results

### **Quantitative assessment of*Fmr1*mRNA and alternative exon 14**

We initially determined the mRNA levels of both total *Fmr1* mRNA and exon 14-containing mRNA by RTqPCR in hippocampus, cerebral cortex, and cerebellum of rats between E19 and P2. The selection of developmental stages and CNS areas has been based on previously published *FMR1* expression and functional studies [7, 38–42]. Total *Fmr1* messages were amplified with primers specific for exons 10 and 11, constituting an amplicon present in all *Fmr1* transcripts. Detection of *Fmr1* exon 14 was possible with a primer annealing on that exon, defining a qPCR product with an upstream primer on exon 13 (Fig. 1A). Among the genes tested for relative normalization of *Fmr1* expression rates, none appeared reliable. While *Ppia* mRNA amount did not significantly differ between the brain areas, it varied widely among developmental stages. The *Actb* and *Gapdh* expression rates varied extensively in both areas and periods (data not shown). Consequently, the amplicon quantification was based on the variability between the samples in view of the reasonable biological sample size (N ≥ 7 for each test group), and the technical triplicates of each one.

In the three analyzed CNS areas, total *Fmr1* mRNA quantity increased from E19 to P2 (*P* < 0.05, *P* < 0.01, and *P* < 0.001, respectively for hippocampus, cerebral cortex, cerebellum), as expected [13, 39, 42–44]. Within that period, we observed a parallel rise in the amount of *Fmr1* messages displaying exon 14 in all the three brain regions (*P* < 0.05, *P* < 0.05, and *P* < 0.01, respectively for hippocampus, cerebral cortex, and cerebellum; Fig. 1B, C, E). These results indicate that the increase of specific exon-14-containing messages from E19 to P2 reflects the global rise in *Fmr1* mRNA amount.

Specific exon junctions in *Fmr1* mRNA lacking exon 14 were examined with novel primer pairs (Fig. 1A). Amplicons 13–15A, 13–15B and 13–15C had a common sense primer sequence hybridizing to exon 13 and one of three antisense, exon junction-spanning primers partially annealing to exon 13 3´ end and the 5’ end of exon 15 immediately downstream of splicing acceptor sites 15A, 15B or 15C, respectively. Between E19 and P2 the amount of 13–15A and 13–15B amplicons significantly increased in hippocampus and cerebellum (respectively, *P* < 0.05 and *P* < 0.01 for both amplicons). Amplicon 13–15C expression significantly increased in cerebellum (*P* < 0.001) but not in hippocampus (*P* > 0.05; Fig. 1B, E). This expression enhancement could reflect an overall increment in total *Fmr1* mRNA quantity and similar exon 14 combinatorial splicing outputs occurring on E19 and P2 in the hippocampus and cerebellum, inferring no differential regulation between those developmental days in each structure. On the other hand, no differences were observed for any 13–15 amplicons (*P* > 0.05) between E19 and P2 in cerebral cortex (Fig. 1C), suggesting that in this brain area exon 14 skipping is regulated separately from overall *Fmr1* mRNA levels.

As there were no differences in 13–15 A, B or C amplicons in cerebral cortex between E19 and P2, the lower levels of exon 14 on E19 inferred that skipping of this exon should be more remarkable in E19 cerebral cortex. For further insights, we compared *Fmr1* expression levels between E19 cerebral cortex and E14 telencephalic vesicles as these are the developmental primordium of the cerebral cortex. Although total *Fmr1* messages did not differ between E14 telencephalic vesicles and E19 cerebral cortex (*P* > 0.05), we detected a decrease in exon 14 amplicon in E19 cerebral cortex when compared to E14 telencephalic vesicle mRNA (*P* < 0.01), while quantities of 13–15A, 13–15B and 13–15C amplicons did not reveal any statistically relevant differences (*P* > 0.05; Fig. 1D). These data further suggested that *Fmr1* mRNA lacking exon 14 should be notable in E19 cerebral cortex. The novel data on E19 cerebral cortex corroborating unchanged levels of any 13–15 amplicons could be explained by specific degradation of *Fmr1* messages lacking exon 14.

### RNA-Seq BLAST validation of the RTqPCR results

**Fig. 2:**
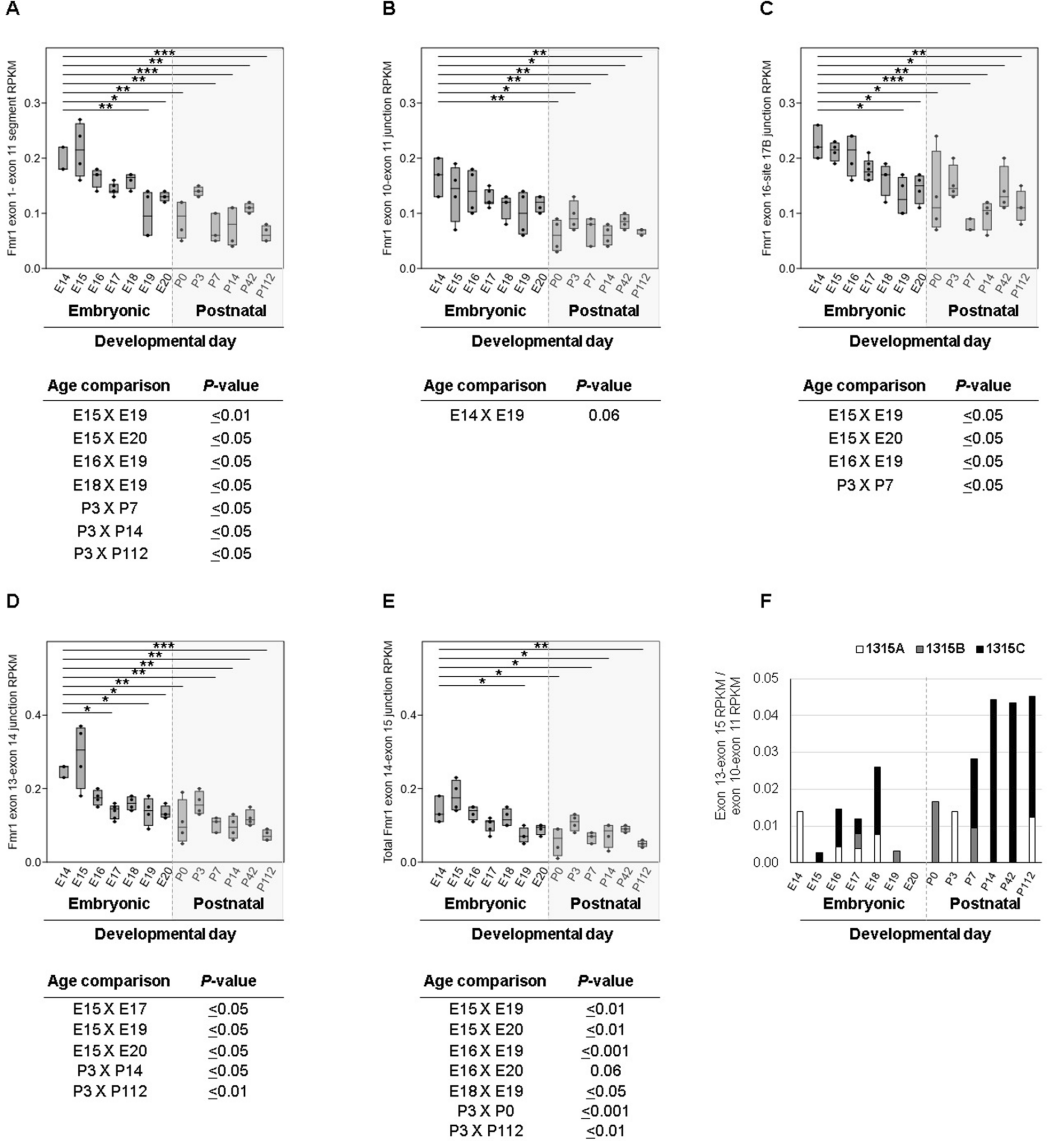
BLAST RNA-Seq RPKM values quantifying* Fmr1* sequences in the rat forebrain. Box plots of RPKM values for BLAST searches with (**A**) *Fmr1* coding sequence from exon 1 through 11 [1–11]; or sequences spanning junctions between (**B**) exons 10 and 11, (**C**) 16 and 17B, (**D**) 13 and 14, (**E**) 14 and the summation of data for the three exon 15 sites. **F** RPKM ratios of 13–15A, 13–15B or 13–15C junction by exon 10 and 11 junction sequence reads. Significant Dunn´s *post-hoc* test results are indicated on graphs by lines with asterisks according to *P* values for comparisons to E14 RPKM values (*P < 0.05; **P < 0.01; ***P < 0.001). Other significant comparisons between embryonic ages or involving P3 are shown below each graph (A–E). N = 4 for each age group, except E14 and P7 (N = 3), and E17 (N = 6)

In view of significantly increased *Fmr1* exon 14 skipping in E19 cerebral cortex, we statistically assessed rat *Fmr1* BLAST RPKM values of 52 forebrain, 49 cerebellum, and 20 whole brain individual RNA-Seq datasets of a single project (PRJEB26889) [45]. Forebrain results are presented first, and whole brain and cerebellum data are discussed afterwards. To certify that short targets of 100-nucleotide length would provide reliable data, our BLAST strategy initially targeted a 1,122-bp *Fmr1* coding sequence comprising the first eleven consecutive and constitutive exons [exons 1–11] or a 100-nucleotide sequence spanning the junction between constitutive exons 10 and 11 (exons 10–11). This initial analysis was also useful to establish the filtering parameters employed in all BLAST searches. Among 13 forebrain ages (E14 to P112), RPKM values were significantly different for exons 1–11 (H (χ^2^) = 43.76, fd = 12; *P* < 0.001) and exon 10–11 junction (H (χ^2^) = 30.74, fd = 12; *P* < 0.01). Paired comparison results were remarkable for the highest *Fmr1* mRNA RPKM values on E14−E18 for both baits, which were significantly different from postnatal ages P0, P7, P14 and P112, whereas on P3 exon 1–11 RPKM values were similar to embryonic ages (Fig. 2A, B). Exon 1–11 or 10–11 RPKM values were similar between each embryonic day from E15 to E18 and P3 (Additional file 1: Table S3).

Sequences of cryptic exon 9a or spanning exon 16–17A junction retrieved no reads for RNA-Seq BLAST searches of any structure. BLAST results for the 16–17B junction sequence showed significant differences among ages (H (χ^2^) = 33.1, fd = 12; *P* < 0.001) similarly to those of constitutive exons (Fig. 2A–C, Additional file 1: Table S3), corroborating 17B as a constitutive splice acceptor site of the *Fmr1* primary transcript in the rat nervous system. Notably, *Fmr1* constitutive exon junction 10–11 had similar RPKM values among embryonic ages, albeit lower levels on E19 than on E14 close to significance (Fig. 2B), whereas baits 1–11 and 16–17B disclosed lower levels on E19 than on E14−E16 (Fig. 2A, C).

As the strategy of BLASTing short sequences spanning exon junctions was validated for *Fmr1* constitutive exons, we pursued the analysis for alternative exons 14 and 15. Sequences spanning any of the three junctions (A, B or C) between exons 14 and 15 yielded more RNA-Seq reads in embryonic forebrain (Additional file 1: Fig. S1), when total *Fmr1* mRNA is higher than on postnatal days (Fig. 2A–C). Usage of the 15C site decreased on E17, E19 and E20 comparatively to other embryonic days, and 14–15A sequences corresponded to nearly 40% of total 14–15 junctions (Additional file 1: Figs. S1, S2).

**Fig. 3:**
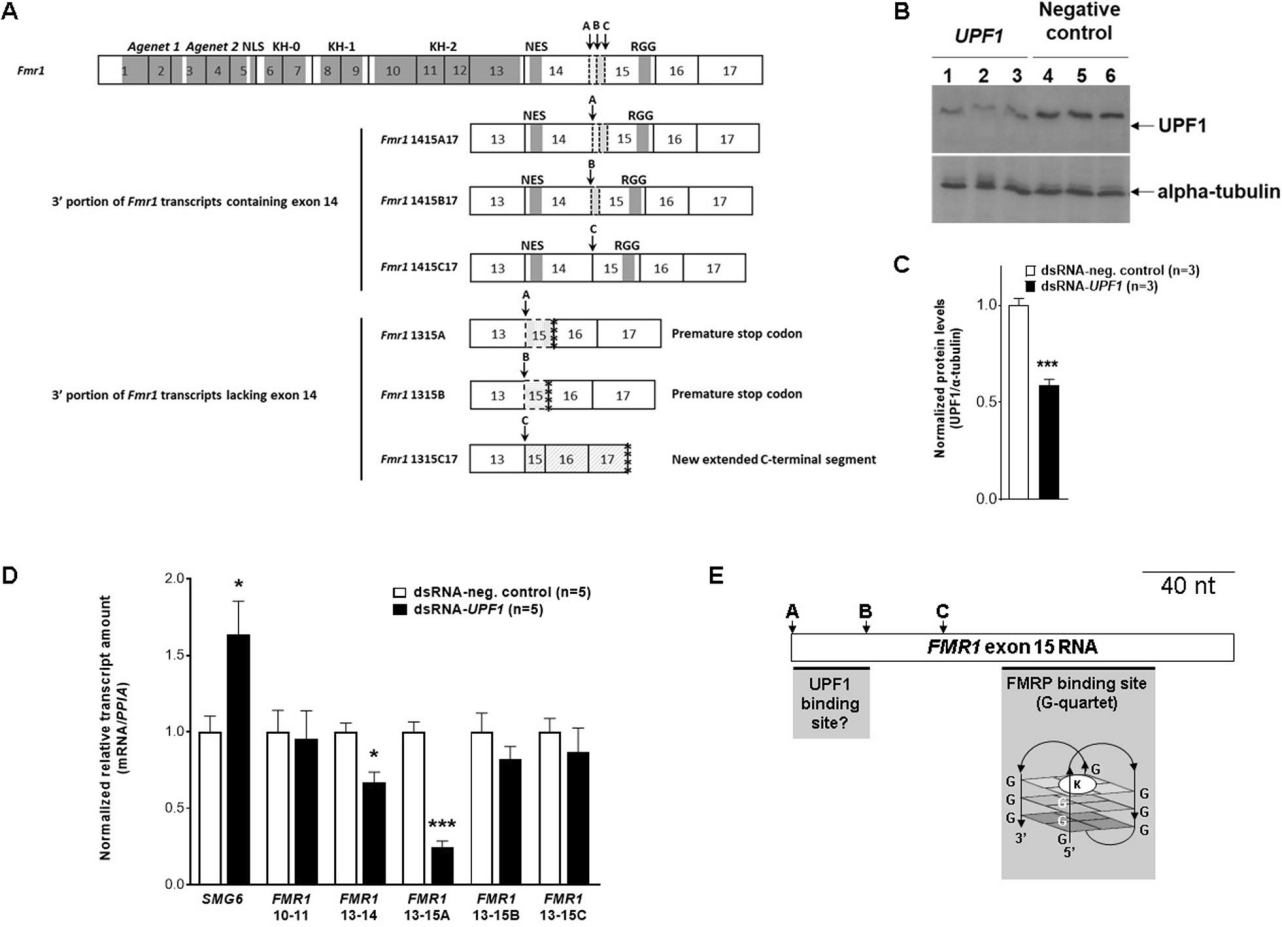
Knocking down *UPF1* expression as a tool for assessing NMD-induced effects on *FMR1* mRNA. **A** Schematic diagrams for *FMR1* gene and transcripts. Full-length *Fmr1* coding exons 1 to 17 (numbered boxes) and FMRP encoded domains, nuclear localization (NLS) and export (NES) signal motifs. *FMR1* transcripts with or without exon 14 and selection of splice acceptor sites 15A, 15B or 15C are partially illustrated (exons 13 to 17). Location of translation termination codons is indicated by asterisks. **B**, **C** Western blot analysis of lysates of HEK293T transfected with *UPF1* or control dsRNA, as indicated, treated with antibodies for UPF1 or α-tubulin, and plot of densitometry intensities. **D** RTqPCR data normalized by *PPIA* for HEK293T cells transfected with *UPF1* or negative control dsRNAs, for *SMG6* (positive control), *FMR1* exons 10 and 11, exons 13 and 14, and amplicons 13–15A, 13–15B and 13–15C. Student´s *t* test: *P* < 0.05 (*), *P* < 0.01 (**), and *P* < 0.001(***). **E** Illustration of *Fmr1* exon 15 sequence RNA (length legend in nucleotide, nt) and location of splice sites A, B and C, as well as the segment able to form a G-quartet structure stabilized by a counterion (K^+^). A suggested location of a UPF1 binding site is indicated between sites 15A and 15B

*Fmr1* exon 14 was assessed by BLASTing RNA sequences spanning its upstream junction with exon 13 (13, 14) or downstream with exon 15 (14, 15). RPKM values varied for junctions 13–14 (H (χ^2^) = 36.06, fd = 12; *P* < 0.001) as well as the summation of exon 14 joined to 15A, 15B and 15C splice sites (H (χ^2^) = 37.51, fd = 12; *P* < 0.001). Early embryonic ages E14 and E15 had higher 13–14 and 14–15 RPKM values than E17, E19 and E20 or P0 and P7–P112 (Fig. 2D, E, Additional file 1: Table S3). Sequences spanning 13–15A, 13–15B and 13–15C junctions retrieved limited read numbers. Of note, although low, 13–15C reads corresponded to 3–5% of total RPKM values for the junction between exons 10 and 11, in P14–P112 forebrain (Fig. 2F). The paucity in 13–15 read retrieval, in particular when exon 14 skipping should be more remarkable (E17, E19, E20; Fig. 2D–F), is further inference for degradation of *Fmr1* messages without exon 14, which are potential NMD targets, as skipping this exon produces PTC (Fig. 3A).

### ***UPF1*knock-down effects on*FMR1*mRNA**

To test the hypothesis that NMD may reduce *Fmr1* exon-14-skipped mRNA levels, we knocked down the expression of a major protein component of that pathway, the regulator of nonsense transcripts 1 (UPF1), in the human cell line HEK293T. We first confirmed the suitability of this cell line to functionally study *FMR1* expression regulation [46] by comparing *Fmr1* mRNA expression levels between non-transfected HEK293T cells and elderly human cerebral cortex (Additional file 1: Material). As expected, the ensemble of *FMR1* transcripts and those specifically expressing exon 14 were all higher in HEK293T cells than in adult human cerebral cortex (Additional file 1: Fig. S3).

Knocking down *UPF1* mRNA in HEK293T cells significantly decreased the UPF1 protein expression (Fig. 3B, C) and increased the mRNA quantity of *SMG6* (Fig. 3D), a recognized target of NMD [47], but did not affect the amount of amplicons assessing exon junctions 10–11, 13–15B or 13–15C (Fig. 3D). However, an unexpected significant decrease in mRNA amount was observed for amplicons addressing the 13–14 and 13–15A junctions (Fig. 3D).

### Whole brain and cerebellum RNA–Seq data sets

RNA-Seq BLAST data of cerebellum and whole brain revealed limited *Fmr1* mRNA expression variation among ages. It is possible that, for this reason, the correlation between significant RPKM differences for distinct *Fmr1* sequence targets was not as extensive as seen for the forebrain. For whole brain, the *Fmr1* 1–11 sequence presented lower RPKM values at E14 or E16 than at E11. Similarly, on E16, there were fewer reads for exon 14–15 junctions (summation of all exon 15 sites) than on E11, E12 or E13, or for exon 14 joined to individual sites 15A or 15C than from E11 to E14 (Additional file 1: Fig. S4), but no remarkable differences for exon 10–11, 16–17B or 13–14 junctions. Of note, cerebellum on E19 and P7 through P112 had lower levels of *Fmr1* messages detected by BLASTing 1–11 sequence, and 14–15, 14–15A or 14–15C junctions (Additional file 1: Fig. 5).

### FMRP expression in E19 and P2 rat brain

**Fig. 4:**
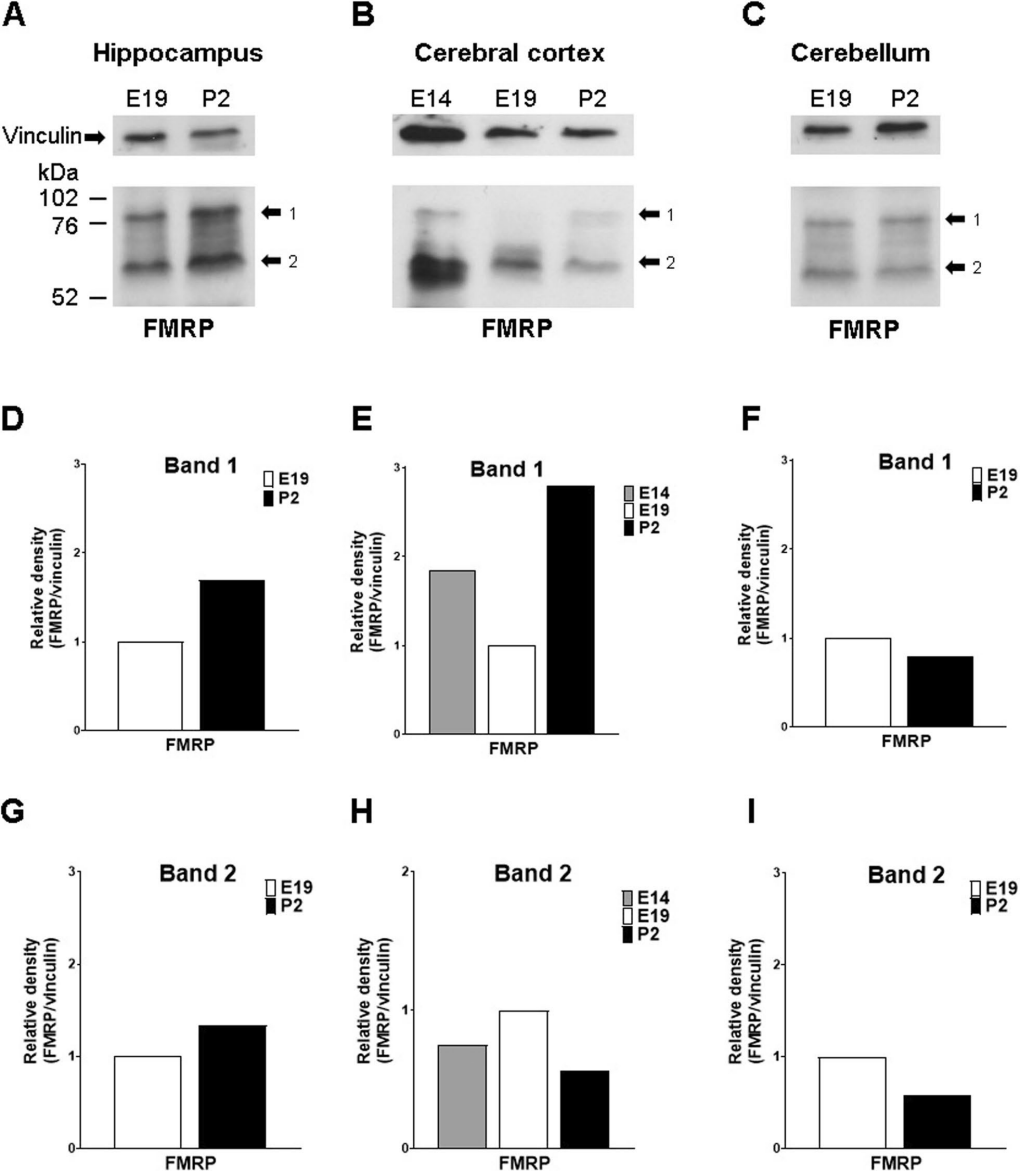
FMRP immunoblot of rat brain structures. **A**–**C** Panels of blots incubated with anti-vinculin or anti-FMRP, as indicated, corresponding to hippocampus (**A**), cerebral cortex (**B**) and cerebellum (**C**). Arrows indicate the blot areas that had the intensity estimated and labeled as bands 1 or 2. **D**–**I** Plots of band 1 (**D**–**F**) or band 2 (**G**–**I**) intensities normalized by the vinculin band intensity for hippocampus (**D** and **G**), cerebral cortex (**E** and **H**) and cerebellum (**F** and **I**)

To verify the overall level of FMRP in the developmental periods and brain areas in which this study disclosed significant changes of total *Fmr1* mRNA amount (Figs. 1,  2), we performed immunoblotting of tissue lysates of pooled rat hippocampus, cerebral cortex or cerebellum on E19 and P2, as well as E14 telencephalic vesicles. Immunoblotting was conducted with an antibody that detects an invariable segment of FMRP N-terminus (2F5-1;[41]. Western blotting analyses have reported FMRP as a group of bands migrating between 60 and 75 kDa [4, 13, 34, 38]. It is not possible to correlate the individual bands on blots to specific FMRP isoforms, because anti-FMRP antibodies detect invariable segments of the protein and FMRP undergoes several posttranslational modifications that alter its migration in gel [28, 29, 34]. However, according to previous transcript assessments [27], it is assumed that the strongest slow-migrating band corresponds to the most frequently expressed FMRP isoform (isoform 7, [22]. In our analysis, we selected two blot areas within the FMRP molecular mass range to estimate the intensity of bands that were collectively denominated as bands 1 and 2, referring to the slow and fast-migrating bands seen on Fig. 4 (A–C), respectively. As normalized by the intensity of the band of the cytoskeleton protein vinculin, obtained by reprobing the same membrane, there was an overall increase in the ratio of either bands 1 or 2 when signals for E19 and P2 hippocampus were compared (Fig. 4A, D and G). For cerebral cortex, the normalized band 1 ratio was lower on E19 than on P2 or comparing E19 to E14 telencephalic vesicles (Fig. 4B and E). Conversely, cerebral cortex band 2 ratio was higher on E19 than on P2 or E14 telencephalic vesicles (Fig. 4B and H). In cerebellum, the ratios for bands 1 and 2 were overall higher on E19 than on P2 (Fig. 4C, F and I).

## Discussion

**Fig. 5:**
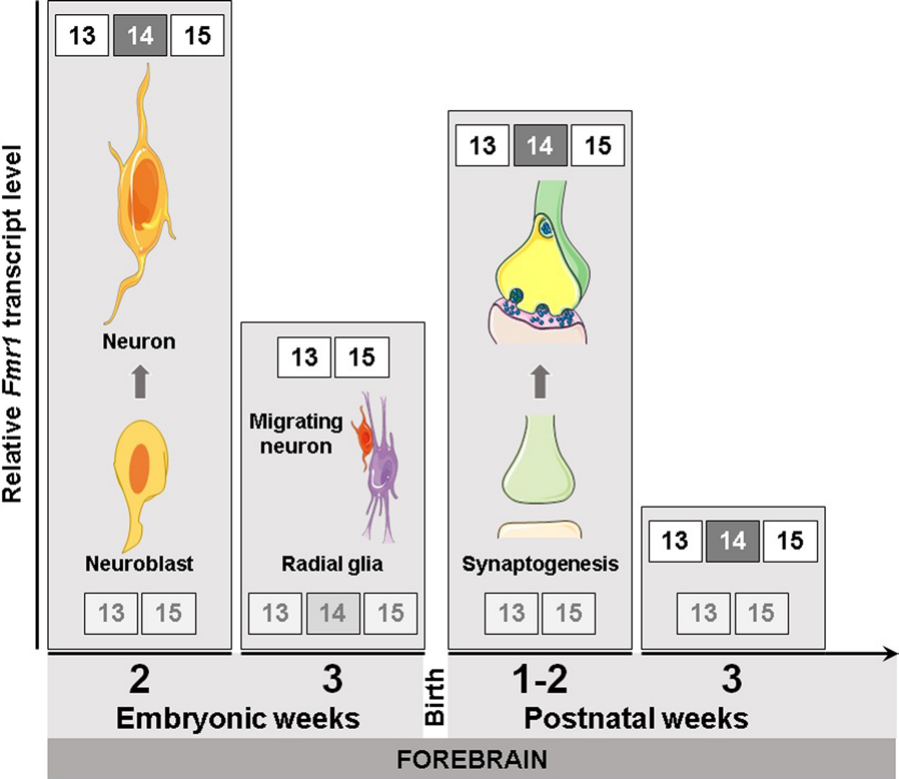
Graphic scheme correlating the summary of the results to known neurodevelopmental aspects of FMRP. The column graphic style of the illustration presents the relative amount of total *Fmr1* transcripts in the forebrain as columns, in a limited period of the development: the last two embryonic weeks (2 and 3) and the first three postnatal weeks (1–2 and 3). According to the data presented here, total *Fmr1* mRNA reaches its highest values on the second embryonic and first postnatal weeks. Low levels of *Fmr1* mRNA are observed in the third embryonic week as well as after the second postnatal week. In each pictured graph column of total *Fmr1* mRNA, the most abundant exon 13 junction is represented on the top, while on the bottom part is the least frequently observed one in a fading color tone. A drawing of the predominant neuronal differentiation process that takes place in each of the illustrated developmental week highlights the first three graph columns. Each process is associated with a developmental period, representing neurogenesis that predominates in embryonic week 2, neuron migration in embryonic week 3; and synaptogenesis starting in postnatal weeks 1 and 2

This descriptive study presents for the first time an extended forebrain and cerebellum developmental study of *Fmr1* mRNA expression. We demonstrate a progressive decline of forebrain *Fmr1* mRNA amount from E14 through P112 except for an increase on P2-P3, and reveal E17 to E20 as a period of increased exon 14 skipping (Figs. 1, 2 and 5). Knocking down *UPF1* expression did not increase the stability of 13–15 junction-containing mRNAs (Fig. 3), suggesting that NMD does not elicit their degradation. Finally, we provide evidence for the *Fmr1* splice site 17B as a constitutive site in the rat brain and developmental period studied, and no recognition of the cryptic exon 9a under these conditions.

Previous studies on *FMR1* alternative splicing focused on specific variable transcripts [21–23, 26, 27, 48]. Brackett et al. [27] conducted quantitative mRNA studies of 12 mouse *Fmr1* variable transcripts, differing in inclusion or exclusion of exons 12 or 14 and selection among splice acceptor sites 15A, 15B, and 15C. Their study revealed that *Fmr1* alternative splicing occurs in 11 structures of the mouse brain, and that messages with exon 12 are relatively increased in E9 brain and cultured neurospheres. Transcripts without exon 14 were the least abundant *Fmr1* messages in whole brains, while the most frequent transcripts contained exon 14, but lacked exon 12 [27], encoding FMRP isoforms 7, 8 and 9 [22]. All variable transcripts were found mostly associated with polyribosomes, inferring that they should be translated [27].

Here, we present a descriptive study of total *Fmr1* and specifically its exon 14 expression along the development of the rat forebrain. The *Fmr1* knockout rat presented by Till et al. [35] provided cross-species validity of multiple cellular phenotypes associated with loss of FMRP in mouse and rat, including elevated basal protein synthesis, abnormal synaptic plasticity, and alterations in the morphology of dendritic spines of hippocampal pyramidal neurons. By contrast, performance in spatial reference memory, reversal learning and delayed matching to place tasks, which were altered in the mouse model, were not in the *Fmr1* knockout rat, indicating rat-specific hippocampal-based memory behaviors in the absence of FMRP. An additional *Fmr1* knockout rat developed by Tian et al. [36] presented impaired long-term synaptic plasticity, hippocampus-dependent learning and social interaction, as well as macroorchidism, similar to fragile X syndrome male patients. Hence, cross-mammalian comparisons of specific gene function in a complex environment such as the brain allow for identification of commonalities as well as specific aspects of molecular, cellular, and physiological phenotypes [35, 36]. It is thus of great importance to address orthologous gene expression patterns in different species, in order to strengthen common molecular aspects likely to contribute to understand the particularities of the pathway.

We individually quantified alternative exon 14 in rat *Fmr1* mRNA, in search for specific time points and brain structures with its significant expression or skipping. The selection of tissues for RTqPCR and RNA-Seq BLAST analyses were based on recognized expression and functional roles of FMRP as a translational regulator in the CNS. In the rodent forebrain, from E14 to E19, there is a considerable development of the cerebral cortex as neurons migrate and assemble most of the cortical lamination [19], whereas E19 through P2 defines a developmental transition including birth when gene expression program switching should take place [49, 50]. In rodent cerebral cortex, hippocampus and cerebellum, FMRP expression reaches its highest levels in the first postnatal week, a period of intense synaptogenesis [39, 42]. The expression of synaptogenic markers, such as synaptophysin and PSD-95, can be observed in the rodent cerebral cortex and hippocampus by the end of the first postnatal week; from then on there is a steep rise in their protein levels [51, 52]. Therefore, a first wave of increasing FMRP steady-state levels precedes that of the synaptogenesis protein markers in the early postnatal days in the hippocampus and cerebral cortex [39, 42]. Later in postnatal development, exposure of young adult rats to sensory (dark-reared animals exposed to light or to complex visual environment, whisker stimulation) or motor (training on motor-skill tasks) inputs increase cerebro-cortical FMRP synthesis [13, 38, 40, 41, 53].

BLAST results were presented after normalization by the *Fmr1* bait sequence length and RNA-Seq data size (RPKM). Transcriptome data size reflects endogenous RNA profile and technical aspects including cell lysis, RNA capture, library construction, sequencing platform procedures and pipeline for sequence alignment to the reference genome. Endogenous mRNA steady state is a product of gene transcription rates by mRNA decay levels. Database analysis of transcriptome lacks in vitro verification of experimental errors by the computer researcher, such as for RNA dosage control. In our process of analyzing RPKM data, we excluded outlier samples due to extremely high numbers of reads for different *Fmr1* sequences (data not shown). Other limitations of RNA-Seq BLASTing concerns the sequencing approach. The analyzed project (PRJEB26889) based on unpaired, 101-nucleotide reads. Naturally, sequencing platforms generating longer reads are more amenable for splice junction detection and quantification; however, so far it is unlikely to retrieve datasets with these characteristics for the mammalian forebrain or cerebellum with the extensive development coverage analyzed here. We believe that, by using our filter variables, read mapping certainty was considerably high, even without paired-end reads. In summary, data normalization, outlier exclusion, target sequence length validation (Fig. 2A–C), high filtering stringency and elevated consistency between RTqPCR and BLAST results are internal control measures that support and strengthen our results. The use of two different rat strains for the *Fmr1* RNA analyses, Wistar rats for RTqPCR studies (Fig. 1) and the Holtzman RD strain in the published RNA-Seq data (Fig. 2; [45], did not affect the final results, depicting similarities between the two groups of results.

Our data demonstrating significant changes in mRNA amount of *Fmr1* alternative exon 14 are indicative of specific developmental splicing regulation. Forebrain *Fmr1* RTqPCR disclosed increased exon 14 skipping on E19. BLAST analysis extended the stage of significant exon 14 skipping to the late embryonic period (E17–E20). Moreover, total *Fmr1* transcripts were higher on P2 than on E19 (Fig. 1). Forebrain BLAST analysis of constitutive exons employing two distinct baits revealed similar levels of total *Fmr1* mRNA on P3 and E14–E15 that were significantly higher than on P7–P14 or P112 (Fig. 2A and C). From P7 onwards *Fmr1* constitutive exons had lower mRNA than embryonic stages (Fig. 2A–C and Additional file 1: Table S3). Although the reduced levels of *Fmr1* mRNA on E19 did not reach significance for the 10–11 exon junction (Fig. 2B), 1–11 and 16–17B exon junction RPKM values were lower on E19 than on E14–E16, and lower on E20 than on E14 or E15 (Fig. 2A and C). Regarding exon 14 in mRNA, a significant reduction on E17–P0 was observed comparatively to E14 or E15, despite unaltered levels on E18 (Fig. 2D–E). The lack of difference, such as on E18, should be explained by experimental variables, e.g. tissue collection procedures, which may impact on mRNA quantification reproducibility.

The data may infer that developmentally increased exon 14 skipping on E17–E20 and possibly consequent degradation of the resulting *Fmr1* mRNA (Fig. 2E) could lead to overall *Fmr1* down-regulation in late embryonic stage. In this developmental transition, the E17 decrease in exon 14 mRNA preceded E19 total *Fmr1* mRNA reduction (Fig. 2). By E17–E20, neurogenesis has decreased in the rat forebrain, and neuronal migration is actively taking place [54]. Thus, it is possible that *Fmr1* mRNA levels decrease as exon 14 is more often skipped and the resulting messages are degraded.

Various lines of evidence indicate a subtle balance of mRNA and protein is necessary during cerebral corticogenesis, involving different levels of regulation of the expression of critical genes [55]. Micro RNAs (miRNA) targeting *Drosophila*, mouse or human *Fmr1* 3’-UTR have been identified and validated. In particular, mouse miR-129 is expressed in neural progenitor cells, cortical and hippocampal neurons. MiR-129-5p targets the murine *Fmr1* mRNA in vitro, reducing its levels as well as of Fmrp [56, 57]. *In utero* electroporation of E14.5 mouse embryos leading to the knockdown of miR-129-5p in the brain decreases the number of progenitor cells in the germinal and intermediate layers and increases neurons in both deep and upper cerebro-cortical layers [57]. Overexpressing *Fmr1* in E14.5 mouse brain similarly reduces the number of cells in the germinal and intermediate layers and increased the number of deep layer but not of the upper layer neurons. Curiously, the knockdown of *Fmr1* on the same embryonic day also reduced the number of cells in the germinal zone and increased the amount of deep layer neurons. Conversely, it increased the number of cells in the intermediate zone and reduced that of the upper layer neurons [57]. It is plausible that the mouse cerebral corticogenesis is sensitive to the Fmrp dose. The co-overexpression of miR-129 and Fmrp in E14.5 mouse brain had intermediate zone cell amounts comparable to the control [57]. Other mouse miRNA, miR-130b and miR-124, have been shown to reduce both *Fmr1* mRNA and Fmrp and affect the proliferation rate of embryonic neural precursor cells [58, 59].

Interestingly, in *Xenopus laevis* tadpoles, the developing optic tectum, the midbrain structure responsible for processing most visual signals in non-mammalian vertebrates, is affected by quantitative alterations of Fmrp. The increase or reduction of Fmrp decreases neural progenitor cell proliferation and/or raises cell death, what may eventually lead to exhaustion of the progenitor cell pool [60]. Hence, the tadpole neurogenesis is sensitive to the Fmrp quantity.

The stability of *FMR1* mRNA can be affected in the late adult-onset neurodegenerative disorder fragile X-associated tremor and ataxia syndrome (FXTAS). FXTAS is due to premutation-sized CGG repeat expansions (55–200 repeats) in the *FMR1* first exon, possibly leading to a gain-of-function nuclear accumulation of the *FMR1* mRNA (reviewed by Suardi and Haddad, 2020 [2]). The expanded trinucleotide repetitive region in the 5’-UTR of the *FMR1* mRNA may assemble into long hairpins that sequester nuclear proteins, including splicing factors [61–63]. Recent work has disclosed an increase in *FMR1* transcript isoform 10, which lacks both exons 12 and 14, in premutated FXTAS patient leukocytes compared to non-FXTAS premutated men or healthy normal allele carriers [64]. It is generally assumed that a splicing factor unbalance modifying the transcript output could in part contribute to the pathophysiology of FXTAS. Moreover, the expression of an antisense noncoding gene overlapping *FMR1* first exon (*ASFMR1*) is upregulated in cells with *FMR1* premutation [64, 65]. The antisense hybridization of *ASFMR1* ncRNA to *FMR1* mRNA 5’ end could function as an additional mechanism to regulate its stability and half-life.

Epigenetic alterations may control transcription and splicing [66]. *FMR1* CGG repeat expansions into full mutations lead to cytosine methylation, epigenetic silencing of the allele and fragile X syndrome. Most efforts to understand the *FMR1* epigenetic marks have been towards the characterization of the epigenetic modifications associated with the gene silencing mechanism leading to fragile X syndrome [67]. Thus, it is yet unknown which chromatin marks regulate *Fmr1* splicing. Interestingly, FMRP has been identified as a trans-acting factor that when bound to *Fmr1* pre-mRNA exon 15 G-quartet can influence the selection of the upstream splice acceptor sites 15B and 15 C by the spliceosome [68].

RNA messages targeted to NMD are expected to increase in quantity upon *UPF1* expression knock-down [47]. As our assay did not provide any evidence for UPF1 triggering the degradation of HEK293T *FMR1* mRNA with 13–15 junctions (Fig. 3D), it is likely that other pathways may account for their decay, e.g., the nuclear exosome [69]. To engage with the substrate, the nuclear exosome first associates with the adaptor complexes nuclear exosome targeting (NEXT) or poly-A tail exosome targeting (PAXT). While NEXT targets non-polyadenylated RNA substrates for decay, PAXT targets polyadenylated RNAs not yet exported to the cytoplasm. They share the helicase MTR4, and each complex, NEXT or PAXT, has a zinc-finger protein of its own, ZCCHC8 and ZFC3H1, respectively. RNA interference of their transcripts has proved as a reliable in vitro system to identify NEXT and PAXT targets [70], and could possibly be employed to assess the decay of the *Fmr1* mRNA without exon 14.

By contrast, knocking down *UPF1* expression in HEK293T cells decreased the levels of *Fmr1* mRNA with splice junctions 13-14 or 13–15A, but not 13–15B or 13–15C (Fig. 3D), suggesting specific message stabilization by UPF1. FMRP has been recently demonstrated to act as NMD repressor by directly interacting with UPF1 bound to mRNA, leading to message stabilization. Accordingly, NMD hyperactivation was observed in *FMR1* knocked-out cells [71]. As FMRP can bind to *FMR1* mRNA by direct association with the G-quartet formed on exon 15 RNA sequence downstream of splice site 15C [68], it is plausible that the cooperation between UPF1 and FMRP stabilizes *FMR1* mRNA in a manner dependent on its G-quartet (Fig. 3E). In this scenario, FMRP would directly bind to *FMR1* mRNA G-quartet and to UPF1. It is unclear if this protein-protein interaction depends on exon junction complexes [71]. It has been reported that UPF1 can directly bind to mRNA binding sites located within or close to structured G-rich sequences [72]. Thus, it is possible that exon 15 sequence between splice sites 15A and 15B immediately upstream of the G-quartet is necessary for UPF1 binding (Fig. 3E). This suggests that the stabilization of *FMR1* message by UPF1 may depend on its optimal binding to FMRP depending on the availability of the full exon 15 sequence in the mRNA. Accordingly, *Fmr1* exon 14 junction with exon 15 in the rat brain was more frequently observed by splice site 15A usage rather than by 15B or 15C (Additional file 1: Fig. S3).

Differences in forebrain *Fmr1* mRNA expression rates (e.g., between P3 and P14) when FMRP levels should be high [42] could be justified by multitier regulation of gene expression, including at the translational level. Curiously, the immunoblot evidence for FMRP rise from E19 to P2 in the hippocampus (Fig. 4A, D and G) parallels the increase of total *Fmr1* mRNA in this structure and developmental frame (Fig. 1B). Likewise, the trend to augmentation in intensity of the cerebral cortex blot band 1 from E19 to P2 (Fig. 4B and E) is comparable to the higher levels of *Fmr1* mRNA on P2 (Fig. 1 C). Moreover, it should not be neglected that cerebellum and postnatal forebrain presented a limited, however detectable, number of *Fmr1* messages with the 13-15C exon junction (Fig. 2F and Additional file 1: Fig. S5I). On Western blot, specifically in the forebrain, a set of bands migrating with nearly 60 kDa, here referred to as band 2, could possibly correspond to FMRP isoforms with no expression of exon 14 (isoforms 4, 5, 6, 10, 11 and 12) with expected molecular masses varying from nearly 50 to 60 kDa [22]. However, one cannot assure the specific correspondence between the bands on blots and FMRP isoforms because they can undergo various posttranslational modifications [28, 29], including limited proteolytic processing by calpain 1 [34]. Based on the translational control premise, the endogenous translation of low levels of 13–15C *Fmr1* mRNA remains a possibility to the generation of nuclear FMRP isoforms with novel C-termini as reported in vitro [34].

The balance of FMRP splice forms expressed in an individual neuron can impact the morphology of that neuron [11]. The relationship between alternative splicing and neuronal morphology and function remains to be elucidated. However, alternative splicing affects whether several crucial motifs are included in the mature protein product. For example, alternative splicing impacts RNA binding through the KH2 domain, which is affected by the choice whether to include exon 12 [25], and through the RGG box, which is nonfunctional in FMRP encoded by transcripts that contain the 14–15C splice choice and absent from all transcripts that lack exon 14 [28, 31, 32]. Similarly, kinase cascades that regulate FMRP function act through serine 499 in mouse, serine 500 in human [28], which is absent from splice forms that lack exon 14 or that use the 14–15B or 14–15C splice sites. Moreover, subcellular and nuclear localization may be impacted by the exclusion of exon 14 [22, 32] which results in completely different C-termini that likely respond to distinct upstream regulatory signals. Alternative splicing thus has the ability to profoundly regulate FMRP localization and function.

## Conclusions

In this study we described that rat *Fmr1* mRNA quantity has its highest levels by mid-gestation, when neurogenesis is actively taking place, and upon birth through the first two postnatal weeks (Fig. 5), a period when synaptogenesis is increasing [11, 13, 39, 42]. *Fmr1* expression down-regulation in the last embryonic week of the rat brain follows an increase in exon-14 skipping (Fig. 5). We suggest that the decrease in the amount of *Fmr1* mRNA in the third embryonic week of the rat forebrain development could be in part mediated by increasing exon 14 skipping. Our data do not support the NMD pathway to elicit the degradation of *Fmr1* mRNA without exon 14. Thus, other triggers should be involved. Alternative exclusion of exon 14 shifting the reading frame may be part of a complex combinatorial scenario when different gene expression regulatory mechanisms should lead to down-regulation of *Fmr1* messages.

## Methods

### Animals

Wistar rats were obtained from the animal facility of the University of São Paulo Medical School (São Paulo, Brazil). Research followed international guidelines for the experimental use of animals, and had a protocol approved by the University of São Paulo, Institute of Biosciences internal ethics review board (077/2008). Male embryos on embryonic (E) days E14 and E19, and male newborn rats on P2 were sacrificed under carbon dioxide saturation. Pup brains were dissected isolating the rostral region of the cerebral cortex, the hippocampus and the cerebellum. For E14 pups, the paired telencephalic vesicles were isolated as they are clearly distinguishable from the ventrolateral invaginations of the neuro-epithelium that will originate the basal ganglia. Male rats were selected based on PCR amplification of the *Tspy* gene of the Y chromosome.

### RNA analysis

Total RNA was isolated from rat brain tissue or HEK293T cells using TRIzol reagent (Thermo Fisher Scientific, Waltham MA), following the manufacturer’s protocol. DNAse I treatment (Ampgrade, 1U/µg of RNA, Thermo Fisher Scientific) was performed for 15 min at room temperature to prevent residual DNA contamination. RNA was quantified by spectrophotometry (NanoDrop, Thermo Fisher Scientific). Two micrograms of DNAse-treated RNA of all samples from experimental groups to be compared were simultaneously reversely transcribed using Superscript™ III First-Strand Synthesis System (Thermo Fisher Scientific) according to the manufacturer’s protocol, followed by a 20-min digestion with RNAseH (Thermo Fisher Scientific) at 37 ^o^C.

PCR primer specificity has been verified by agarose gel electrophoresis of RT-PCR products. Amplicons displaying a single band on gel were used in quantitative analyses. Quantitative transcript analyses were performed by a Rotor-Gene 3000 real time PCR equipment (Corbett Research, Concord, NSW, Australia), as previously described [73]. Optimal conditions were determined using a five-point, two-fold cDNA and primer dilution curve for each amplicom. Each qPCR reaction contained 12.5 ng of reverse transcribed RNA, specific primers at 200 nM (Additional file 1: Table S1) and SYBR Green PCR Master Mix (Applied Biosystems, Foster City, CA), following the manufacturer’s conditions. Samples in the absence of cDNA or with RNA (no reverse transcription) were included as negative controls. A dissociation curve was acquired to confirm product specificity and the absence of primer dimers. Relative transcript amount quantification was calculated from three technical replicates, as previously described [74, 75]. Graph design and statistical analyses were performed using Graphpad Prism V6 (GraphPad Software, La Jolla CA). Two-tailed Student’s t-test was used to analyze transcript amount between the two stages: E19 vs. P2, or E19 vs. E14, for each brain area, with significance set at *P* < 0.05. Data were expressed as mean quantity values ± standard error of the mean.

### RNA-Seq data search

Searching the sequence read archive (SRA) database at the National Center for Biotechnology Information (NCBI, Bethesda, MD) for RNA-Seq datasets of rat brain retrieved the project PRJEB26889 of transcriptome analysis of male and female Holtzman SD rat, which includes data of whole brain (E11, E12, E13, E14, E15 and E16), forebrain and cerebellum (E14, E15, E16, E17, E18, E19, E20, P0, P3, P7, P14, P42 and P112; Additional file 1: Table S2). Cerebellum from E14 to E18 corresponded to the prepontine hindbrain-enriched brain region [45]. Sequencing data of each sample were individually submitted to BLAST (Basic Local Alignment Search Tool; NCBI, Bethesda MD) against *Fmr1* sequences: (i) 100-base sequence spanning exon junctions, equally distributed between the two exons (50:50); or (ii) the coding sequence encompassing constitutive exons 1 to 11 (1,122 bases). For each BLAST search, the following variables were allowed: retrieval of maximum of 20,000 sequences, full identity (100%), and E-values (4E–60 to 4E–23). The number of reads was converted to reads per kilobase per million (RPKM). Four outliers (P7 forebrain, E16, E17 and E19 cerebellum, one each) have been removed because higher number of reads have been retrieved for different *Fmr1* sequences suggesting more elevated initial RNA quantities. All the available remaining samples were included in this study, averaging four samples in individual age-tissue groups, except for forebrain on E14 (N = 3), E17 (N = 6) and P7 (N = 3), and cerebellum on E16 (N = 3), E17 (N = 3), E18 (N = 2), E19 (N = 3) and P0 (N = 5). Due to non-normal distribution of RPKM values, we employed the Kruskal-Wallis non-parametric H-test for RPKM median comparison for each structure among all age points. The numbers of classes (k) were the analyzed age points (k = 13 for forebrain and cerebellum, and k = 6 for whole brain). When the null hypothesis was rejected (*P* < 0.05), Dunn´s *post-hoc* was used to compare RPKM median values in age pairs, with significance if *P* < 0.05.

### Cell culture and transfection

The transformed human embryonic kidney cell line (HEK293T) was cultured at 37 ^o^C and 5% CO_2_ humidified atmosphere in Dulbecco´s modified essential medium (DMEM) supplemented with fetal bovine serum at 10% and antibiotics. Knocking-down of human *UPF1* mRNA transcription by RNA interference was conducted in six-well plates with double-stranded RNA (dsRNA) targeting *UPF1* or a negative control dsRNA (Integrated DNA Technologies, Coralville IA; Additional file 1: Table S1; [47]) at 50 nM. Transfection was performed with lipofectamine 2000 according to the manufacturer’s conditions (Thermo Fisher Scientific, Waltham MA). Cells were harvested 72 h after transfection for RNA isolation and RTqPCR, or protein isolation and immunoblotting.

### Immunoblotting

HEK293T cells or dissected brain tissue were washed in phosphate-buffered saline (PBS) and homogenized in RIPA lysis buffer (50 mM Tris, pH 7.4, 150 mM NaCl, 1% NP-40, 0.5% sodium deoxycholate, 0.1 sodium dodecylsulfate, 2 mM EGTA, protease inhibitors (Complete Ultra tablets, Roche, Germany), and phosphatase inhibitors (1 mM sodium fluoride and 1 mM sodium orthovanadate)] for 30 min., at 4 C, and then centrifuged at 16,000*g*, for 30 min., at 4 C. Equal amounts of protein (30 µg) were submitted to 10% SDS-PAGE and transferred to a nitrocellulose filter (45 μm, BioRad, Hercules, CA). The membrane was blocked for one hour in 1% casein (Millipore, Temecula CA) in TBS-T (25 mM Tris, pH 8.0, 140 mM NaCl, 0.1% tween-20). Antibodies for UPF1 (Cell Signaling Technology, Danvers, MA), FMRP residues 1-204 (2F5-1, [41]; Iowa Developmental Studies Hybridoma Bank, Iowa City, IA), alpha-tubulin (clone DM1A, Sigma-Aldrich, Saint Louis, MO) or vinculin (EPR8185, Abcam, Cambridge, UK) were diluted in TBS-T, 2% globulin-free bovine serum albumin (Jackson Immunoresearch, West Grove, PA) and were incubated for one hour at room temperature. After washing in TBS-T buffer, membranes were incubated for 1 h with anti-mouse or anti-rabbit secondary antibodies conjugated to horseradish peroxidase (GE, Piscataway, NJ). A chemiluminescent detection method (ECL prime, GE, Piscataway, NJ) was employed to detect activity of the peroxidase on an X-ray film (GE, Piscataway, NJ). For band intensity quantification, exposed and developed films were scanned in ImageQuant 300 (GE, Piscataway, NJ) and analyzed with ImageJ software [76]. Relative optical density was calculated by dividing the densitometry of the selected FMRP bands by the respective vinculin control value.

## Supplementary Information

**Additional file 1.** Supplementary human subjects and methods. Supplementary references. Oligonucleotide sequences employed in RT-qPCR and PCR for rat or human nucleic acid targets, or dsRNA for RNA interference. Accession numbers of sequence read archive (SRA) database project PRJEB26889 rat RNA-Seqs employed for *Fmr1* BLAST. Significant Dunn´s *post hoc* test results for age pair comparisons of RPKM values obtained in BLAST searches addressing total *Fmr1* or exon 14. Plot of RPKM values obtained by BLAST searches of *Fmr1* sequences spanning junctions between exon 14 and usage of splice site 15A, 15B and 15C. Plot of RPKM ratio of exon 14/exon 15 splice site junction by the summation of all exon 14-exon 15 junctions. *FMR1* mRNA RTqPCR of human aging cerebral cortex and non-transfected HEK293T cells. RPKM values quantifying *Fmr1* sequences in the rat whole brain. RNA-Seq RPKM values quantifying *Fmr1* sequences in the rat cerebellum.

## Data Availability

All data generated or analyzed during this study are included in this manuscript and its additional file.

## Abbreviations

BLAST: Basic Local Alignment Search Tool
CNS: Central nervous system
E: Embryonic day
*FMR1*: FMRP translational regulator 1 gene
FMRP: Synaptic functional regulator FMR1 protein
KH: hnRNP K homology domain
NMD: Nonsense-mediated mRNA decay
P: Postnatal day
PTC: Premature translation termination codon
RGG: Arginine glycine glycine
RPKM: Reads per kilobase per million bases
RTqPCR: Quantitative polymerase chain reaction of reversely transcribed RNA
*SMG6*: SMG6 nonsense mediated mRNA decay facto
*UPF1*: Regulator of nonsense transcripts 1
