# Supplementary material for: Fmr1 exon 14 skipping in late embryonic development of the rat forebrain
Source: BMC Neurosci. 2022 May 31;23:32. doi: 10.1186/s12868-022-00711-1 (PMC9158170; doi:10.1186/s12868-022-00711-1)

Additional file

Supplementary Human Subjects & Methods

Human Subjects

Frozen samples of human postmortem middle frontal gyrus were obtained from the Biobank for Aging Studies (BAS) from the University of Sao Paulo (1). Exclusion criteria for BAS were: (1) brain tissue unsuitable for neuropathological analyses (e.g., cerebrospinal fluid pH<6.5, or major acute brain lesions, such as hemorrhages or tumors); and (2) inconsistent clinical data provided by the informant. Tissue was resected from seven human male subjects whose cause of death had not been brain-related. Age of death varied between 65 and 80 years (mean 71 years). Informed consents were signed by next-of-kin. A knowledgeable informant was interviewed to obtain the deceased's past clinical history using a semi-structured interview, which was previously validated for postmortem use (2, 3). All subjects lacked a history of ischemic cerebral lesion or cognitive decline and had a Clinical Dementia Rating Scale (CDR (4)) = 0. AD-neuropathology was evaluated using the Consortium to Establish a Registry for Alzheimer's disease (CERAD (5)) criteria for neuritic plaques, and the Braak and Braak (BB) score (6) for neurofibrillary tangles (NFT). Cerebrovascular lesions were evaluated macroscopically and microscopically in hematoxylin and eosin stained slides, as reported previously. All subjects had none or low AD neuropathological changes and lacked non-AD neuropathological lesions.

Nucleic acid sequences

Nucleic acid and protein sequence accession numbers are Reference Sequences (RefSeq) retrieved at the National Center for Biotechnology Information (*NCBI*, http://www.ncbi.nlm.nih.gov/nucleotide, Bethesda, MD, USA). Nucleotide RefSeqs are listed on Supplementary Table 1, in which oligonucleotide sequences are also presented.

Supplementary References

1. Grinberg LT, Ferretti RE, Farfel JM, Leite R, Pasqualucci CA, Rosemberg S, et al. Brain bank of the Brazilian aging brain study group - a milestone reached and more than 1,600 collected brains. Cell Tissue Bank. 2007;8(2):151-62.

2. Ferretti REdL, Damin AE, Brucki SMD, Morillo LS, Perroco TR, Campora F, et al. Post-Mortem diagnosis of dementia by informant interview Dementia and Neuropsychologia. 2010;4(2):138-44.

3. Ferretti-Rebustini RE, Balbinotti MA, Jacob-Filho W, Rebustini F, Suemoto CK, Pasqualucci CA, et al. Validity of the Katz Index to assess activities of daily living by informants in neuropathological studies. Rev Esc Enferm USP. 2015;49(6):946-52.

4. Morris JC. The Clinical Dementia Rating (CDR): current version and scoring rules. Neurology. 1993;43(11):2412-4.

5. Mirra SS, Heyman A, McKeel D, Sumi SM, Crain BJ, Brownlee LM, et al. The Consortium to Establish a Registry for Alzheimer's Disease (CERAD). Part II. Standardization of the neuropathologic assessment of Alzheimer's disease. Neurology. 1991;41(4):479-86.

6. Braak H, Braak E. Neuropathological stageing of Alzheimer-related changes. Acta Neuropathol. 1991;82(4):239-59.

7. Cardoso-Moreira M, Halbert J, Valloton D, Velten B, Chen C, Shao Y, et al. Gene expression across mammalian organ development. Nature. 2019;571(7766):505-9.

Supplementary Tables

Supplementary Table 1: Oligonucleotide sequences employed in RT-qPCR and PCR as forward (F) and reverse (R) primers for rat or human nucleic acid targets, or dsRNA for RNA interference

	Oligonucleotide Name	Oligonucleotide Sequence (5'-3')	NCBI RefSeq
**Rat sequences**	*Fmr1* (F)	AGTTGTGAGGGTGAGGATCG	NM_052804.2
	Fmr1 (R)	TTGGAAGGTAGGGAACTTGG	
	Exon 14-Fmr1 (F)	AGTTCTAGGGTAAGGGTGAGGAG	
	Exon 14-Fmr1 (R)	CCTTCTTTCTGATCGTAGCTTAA	
	Exon 13-Fmr1 (F)	GTGGGAACAAAAGACAGCATCG	
	*Exon 13/15A-Fmr1** (R)	AGAATTAGTTC<u>CTTTAAGTAGTTCAGGTG</u>	
	*Exon 13/15B-Fmr1** (R)	TGGTCAATT<u>CTTTAAGTAGTTCAGGTG</u>	
	*Exon 13/15C-Fmr1** (R)	CTGTTGGAG<u>CTTTAAGTAGTTCAGG</u>	
	Ppia (F)	AATGCTGGACCAAACACAAA	NM_017101.1
	Ppia (R)	CCTTCTTTCACCTTCCCAAA	
	Actb (F)	CGTTGACATCCGTAAAGACC	NM_031144.3
	Actb (R)	GCCACCAATCCACACAGA	
	Gapdh (F)	ATGGTGAAGGTCGGTGTG	NM_017008.4
	Gapdh (R)	GAACTTGCCGTGGGTAGA	
	TSPY-1S	ACAATCCTGGGTTGAGGATG	NM_022923.1
	TSPY-1A	GTGCACACAGCCAGTTGAAG	
**Human sequences**	*FMR1* (F)	GCTGATTCAGGAGATTGTGGA	NM_002024.5
	FMR1 (R)	CTGGGGCATTAGGTCCAAC	
	FMR1-exon 14 (F)	GTGGGAACAAAGGACAGCATC	
	FMR1-exon 14 (R)	TAATCTCTCCAAACGCAACTG	
	FMR1-exon 13 (F)	GTGGGAACAAAGGACAGCATCG	
	FMR1-1315A-H* (R)	CAGAATTAGTTC<u>CTTTAAATAGTTCAGGTG</u>	
	FMR1-1315B-H* (R)	GTGGTCAGATT<u>CTTTAAATAGTTCAGGTG</u>	
	FMR1-1315C-H* (R)	CTGTTGGAG<u>CTTTAAATAGTTCAGGTG</u>	
	HPPIA (F)	AATGCTGGACCCAACACAAA	NM_021130.4
	HPPIA (R)	GGCCTCCACAATATTCATGC	
	SMG6 (F)	TTAAGGAGTCCGCCAAAGC	NM_017575.5
	SMG6 (R)	TGATGTCGTCTTGGTCATCC	
	dsRNA UPF1	rGrArU rGrCrA rGrUrU rCrCrG rCrUrC rCrArU rUTT<br>rArArU rGrGrA rGrCrG rGrArA rCrUrG rCrArU rCTT	NM_001297549.2
	dsRNA Negative control	rGrUrA rCrCrU rGrArC rUrArG rUrCrG rCrArG rArArG<br>rUrCrU rGrCrG rArCrU rArGrU rCrArG rGrUrA rCrGrG	Does not apply

*Exon junction-spanning oligonucleotides annealing at the *Fmr1* exon 13 (underlined) and exon 15 (not underlined).

Supplementary Table 2: Accession numbers of sequence read archive (SRA) database project PRJEB26889 rat RNA-Seqs against which *Fmr1* BLAST has been performed (7).

Tissue	Sex	Age	SRA accession Number
Whole brain	MALE	E11	ERX2608627
Whole brain	MALE	E11	ERX2608625
Whole brain	FEMALE	E11	ERX2608621
Whole brain	FEMALE	E11	ERX2608623
Whole brain	MALE	E12	ERX2608629
Whole brain	MALE	E12	ERX2608631
Whole brain	FEMALE	E12	ERX2608637
Whole brain	FEMALE	E12	ERX2608641
Whole brain	MALE	E13	ERX2608649
Whole brain	MALE	E13	ERX2608659
Whole brain	FEMALE	E13	ERX2608645
Whole brain	FEMALE	E13	ERX2608655
Whole brain	MALE	E14	ERX2608669
Whole brain	MALE	E14	ERX2608673
Whole brain	FEMALE	E14	ERX2608661
Whole brain	FEMALE	E14	ERX2608665
Whole brain	MALE	E15	ERX2608689
Whole brain	MALE	E15	ERX2608693
Whole brain	FEMALE	E15	ERX2608691
Whole brain	FEMALE	E15	ERX2608696
Whole brain	MALE	E16	ERX2608718
Whole brain	FEMALE	E16	ERX2608716
Whole brain	FEMALE	E16	ERX2608722
Whole brain	FEMALE	E16	ERX2608725
Forebrain	MALE	E14	ERX2608677
Forebrain	MALE	E14	ERX2608680
Forebrain	FEMALE	E14	ERX2608682
Forebrain	MALE	E15	ERX2608701
Forebrain	MALE	E15	ERX2608703
Forebrain	FEMALE	E15	ERX2608705
Forebrain	FEMALE	E15	ERX2608708
Forebrain	MALE	E16	ERX2608730
Forebrain	MALE	E16	ERX2608732
Forebrain	FEMALE	E16	ERX2608734
Forebrain	FEMALE	E16	ERX2608736
Forebrain	MALE	E17	ERX2608745
Forebrain	MALE	E17	ERX2608750
Forebrain	FEMALE	E17	ERX2608747
Forebrain	FEMALE	E17	ERX2608752
Forebrain	FEMALE	E17	ERX2608762
Forebrain	FEMALE	E17	ERX2608764
Forebrain	MALE	E18	ERX2608789
Forebrain	MALE	E18	ERX2608790
Forebrain	FEMALE	E18	ERX2608782
Forebrain	FEMALE	E18	ERX2608783
Forebrain	MALE	E19	ERX2608795
Forebrain	MALE	E19	ERX2608797
Forebrain	FEMALE	E19	ERX2608791
Forebrain	FEMALE	E19	ERX2608793
Forebrain	MALE	E20	ERX2608815
Forebrain	MALE	E20	ERX2608824
Forebrain	FEMALE	E20	ERX2608817
Forebrain	FEMALE	E20	ERX2608823

Forebrain	MALE	P0	ERX2608839
Forebrain	FEMALE	P0	ERX2608944
Forebrain	FEMALE	P0	ERX2608948
Forebrain	FEMALE	P0	ERX2608951
Forebrain	MALE	P3	ERX2608870
Forebrain	MALE	P3	ERX2608876
Forebrain	FEMALE	P3	ERX2608864
Forebrain	FEMALE	P3	ERX2608882
Forebrain	MALE	P7	ERX2608897
Forebrain	MALE	P7	ERX2608894
Forebrain	FEMALE	P7	ERX2608888
Forebrain	MALE	P14	ERX2608920
Forebrain	MALE	P14	ERX2608917
Forebrain	FEMALE	P14	ERX2608914
Forebrain	FEMALE	P14	ERX2608911
Forebrain	MALE	P42	ERX2608935
Forebrain	MALE	P42	ERX2608939
Forebrain	FEMALE	P42	ERX2608943
Forebrain	FEMALE	P42	ERX2608947
Forebrain	MALE	P112	ERX2608971
Forebrain	MALE	P112	ERX2608967
Forebrain	FEMALE	P112	ERX2608963
Forebrain	FEMALE	P112	ERX2608959
Cerebellum	MALE	E14	ERX2608678
Cerebellum	MALE	E14	ERX2608681
Cerebellum	FEMALE	E14	ERX2608679
Cerebellum	FEMALE	E14	ERX2608683
Cerebellum	MALE	E15	ERX2608702
Cerebellum	MALE	E15	ERX2608704
Cerebellum	FEMALE	E15	ERX2608706
Cerebellum	FEMALE	E15	ERX2608709
Cerebellum	MALE	E16	ERX2608731
Cerebellum	MALE	E16	ERX2608733
Cerebellum	FEMALE	E16	ERX2608735
Cerebellum	FEMALE	E16	ERX2608737
Cerebellum	MALE	E17	ERX2608746
Cerebellum	MALE	E17	ERX2608751
Cerebellum	FEMALE	E17	ERX2608748
Cerebellum	FEMALE	E18	ERX2608781
Cerebellum	FEMALE	E18	ERX2608784
Cerebellum	MALE	E19	ERX2608798
Cerebellum	MALE	E19	ERX2608796
Cerebellum	FEMALE	E19	ERX2608794
Cerebellum	FEMALE	E19	ERX2608792
Cerebellum	MALE	E20	ERX2608833
Cerebellum	MALE	E20	ERX2608816
Cerebellum	FEMALE	E20	ERX2608818
Cerebellum	FEMALE	E20	ERX2608825
Cerebellum	MALE	P0	ERX2608840
Cerebellum	MALE	P0	ERX2608863
Cerebellum	FEMALE	P0	ERX2608845
Cerebellum	FEMALE	P0	ERX2608849
Cerebellum	FEMALE	P0	ERX2608852
Cerebellum	MALE	P3	ERX2608871
Cerebellum	MALE	P3	ERX2608877
Cerebellum	FEMALE	P3	ERX2608865
Cerebellum	FEMALE	P3	ERX2608883
Cerebellum	MALE	P7	ERX2608898

Cerebellum	MALE	P7	ERX2608895
Cerebellum	FEMALE	P7	ERX2608892
Cerebellum	FEMALE	P7	ERX2608889
Cerebellum	MALE	P14	ERX2608921
Cerebellum	MALE	P14	ERX2608918
Cerebellum	FEMALE	P14	ERX2608915
Cerebellum	FEMALE	P14	ERX2608912
Cerebellum	MALE	P42	ERX2608936
Cerebellum	MALE	P42	ERX2608940
Cerebellum	FEMALE	P42	ERX2608944
Cerebellum	FEMALE	P42	ERX2608948
Cerebellum	MALE	P112	ERX2608972
Cerebellum	MALE	P112	ERX2608968
Cerebellum	FEMALE	P112	ERX2608964
Cerebellum	FEMALE	P112	ERX2608960

Supplementary Table 3: Significant Dunn´s *post hoc* test results for age pair comparisons of RPKM values obtained in BLAST searches addressing total *Fmr1* or exon 14

***Fmr1* exon 1-exon 11 segment**		***Fmr1* exon 10-exon 11 junction**		***Fmr1* exon 16-site 17B junction**		***Fmr1* exon 13-exon 14 junction**		***Total Fmr1* exon 14-exon 15 junction**	
**Age**	**P-value**	**Age**	**P-value**	**Age**	**P-value**	**Age**	**P-value**	**Age**	**P-value**
E15XP0	≤0.01	E15XP0	≤0.05	E15XP0	≤0.05	E15XP0	≤0.01	E15XP0	≤0.001
E15XP7	≤0.01	E15XP7	≤0.05	E15XP7	≤0.001	E15XP7	≤0.01	E15XP7	≤0.01
E15XP14	≤0.01	E15XP14	≤0.01	E15XP14	≤0.01	E15XP14	≤0.001	E15XP14	≤0.01
E15XP42	≤0.01	E15XP112	≤0.05	E15XP42	≤0.05	E15XP42	≤0.01	E15XP42	≤0.05
E15XP112	≤0.001	E16XP0	≤0.05	E15XP112	≤0.01	E15XP112	≤0.001	E15XP112	≤0.001
E16xP0	≤0.01	E16XP7	≤0.05	E16XP0	≤0.05	E16XP0	≤0.05	E16XP0	≤0.01
E16XP7	≤0.01	E16XP14	≤0.01	E16XP7	≤0.01	E16XP7	≤0.05	E16XP7	≤0.01
E16XP14	≤0.01	E16XP42	0.07(*)	E16XP14	≤0.01	E16XP14	≤0.05	E16XP14	≤0.05
E16XP42	≤0.05	E16XP112	≤0.01	E16XP42	≤0.05	E16XP42	0.06(*)	E16XP42	≤0.05
E16XP112	≤0.01	E17XP0	≤0.01	E16XP112	≤0.01	E16XP112	≤0.01	E16XP112	≤0.001
E17xP0	≤0.05	E17XP7	≤0.05	E17XP7	≤0.01	E17XP112	≤0.05	E17XP0	0.06(*)
E17XP7	≤0.05	E17XP14	≤0.01	E17XP14	≤0.05	E18XP7	0.06(*)	E17XP112	≤0.05
E17XP14	≤0.05	E17XP42	0.07(*)	E17XP112	≤0.05	E18XP14	≤0.05	E18XP0	≤0.05
E17XP112	≤0.01	E17XP112	≤0.01	E18XP7	≤0.05	E18XP112	≤0.01	E18XP7	≤0.05
E18xP0	≤0.05	E18XP0	≤0.05	E18XP14	≤0.05	E19XP112	≤0.05	E18XP112	≤0.01
E18XP7	≤0.01	E18XP14	≤0.05			E20XP112	0.07(*)		
E18XP14	≤0.01	E18XP112	≤0.05						
E18XP42	≤0.05	E20XP0	≤0.05						
E18XP112	≤0.01	E20XP14	≤0.05						
		E20XP112	≤0.05						

(*)Close to statistical significance

Supplementary figure legends

Supplementary Figure 1: Box plots of RPKM values obtained by BLAST searches of *Fmr1* sequence against RNA-Seq data of rat forebrain on embryonic days E14 through E20, and postnatal days P0, P3, P7, P14, P42 and P112. BLAST baits consisted of *Fmr1* sequences spanning junctions between exon 14 and usage of splice site 15A (A), 15B (B) and 15C (C). Asterisks: Dunn´s *post-hoc* test: * P<0.05; ** P<0.01; *** P<0.001. N = 4 for each age group, except E14 and P7 (N=3), and E17 (N=6).

Supplementary Figure 2: Plot of RPKM ratio between exon 14/exon 15 splice site junction and the summation of all exon 14-exon 15 junctions, according to reads for sequences spanning junctions between exon 14 and sites 15A, 15B and 15C in whole brain (A), forebrain (B) and cerebellum (C).

Supplementary Figure 3: (A) Schematic diagrams for *FMR1* gene and transcripts. The first line illustrates full-length *Fmr1* coding exons 1 to 17 (numbered boxes), and encoded FMRP domains, as well as nuclear localization (NLS) and export (NES) signals. Alternative exons are indicated as white boxes and possible *FMR1* splicing outputs are indicated as inclusion (three top lines) or exclusion (three bottom lines) of exon 14, and usage of splice acceptor sites 15A, 15B or 15C. Locations of translation termination codons are indicated by asterisks. (B) *FMR1* mRNA RTqPCR of human aging cerebral cortex and non-transfected HEK293T cells. Amplicons follow those presented in Figure 1A. Student´s *t* test: P<0.001(***).

Supplementary Figure 4: BLAST RNA-Seq RPKM values quantifying *Fmr1* sequences in the rat whole brain. Box plots of RPKM values obtained by BLAST searches of *Fmr1* sequences against RNA-Seq data of rat whole brain on embryonic days E11, E12, E13, E14, E15 or E16. BLAST baits consisted of (A) *Fmr1* coding sequence from exon 1 through 11; or sequences spanning junctions between (B) exons 10 and 11, (C) 16 and 17B, (D) 14 and 15A, (E) 14 and 15B, (F) 14 and 15C, (G) 13 and 14, (H) 14 and the summation of data for the three exon 15 sites. (I) RPKM ratios between13-15A, 13-15B or 13-15C junction normalized by exon 10 and 11

junction sequence read. Dunn´s *post-hoc* test: * *P*<0.05; ** *P*<0.01; *** *P*<0.001. N = 4 for each age group.

Supplementary Figure 5: BLAST RNA-Seq RPKM values quantifying *Fmr1* sequences in the rat cerebellum. Box plots of RPKM values obtained by BLAST searches of *Fmr1* sequences against RNA-Seq data of rat cerebellum on embryonic days E14 through E20, and postnatal days P0, P3, P7, P14, P42 and P112. BLAST baits consisted of (A) *Fmr1* coding sequence from exon 1 through 11; or sequences spanning junctions between (B) exons 10 and 11, (C) 16 and 17B, (D) 14 and 15A, (E) 14 and 15B, (F) 14 and 15C, (G) 13 and 14, (H) 14 and the summation of data for the three exon 15 sites. (I) RPKM ratios between13-15A, 13-15B or 13-15C junction normalized by exon 10 and 11 junction sequence read. Dunn´s *post-hoc* test: * *P*<0.05; ** *P*<0.01; *** *P*<0.001. N=4 for each age group, except E18 (N=2), E16, E17 and E19 (N=3), and P0 (N=5).

Supplementary Figures

Supplementary Figure 1

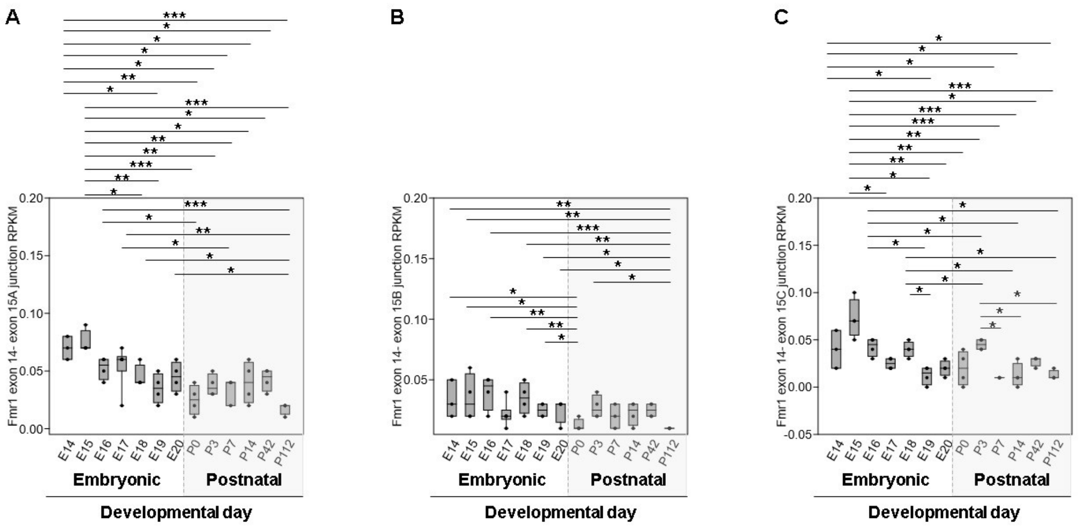

Supplementary Figure 2

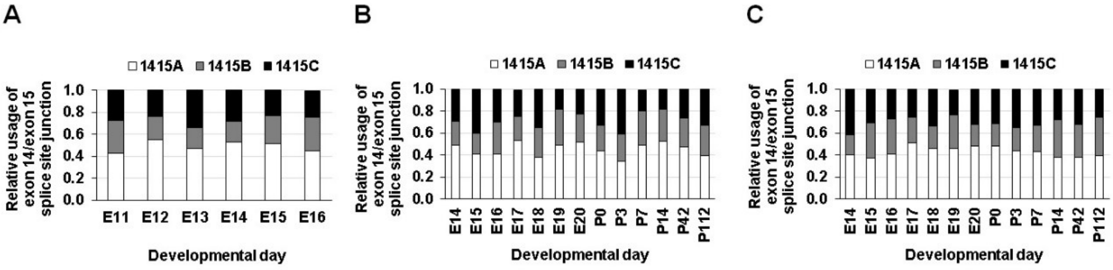

Supplementary Figure 3

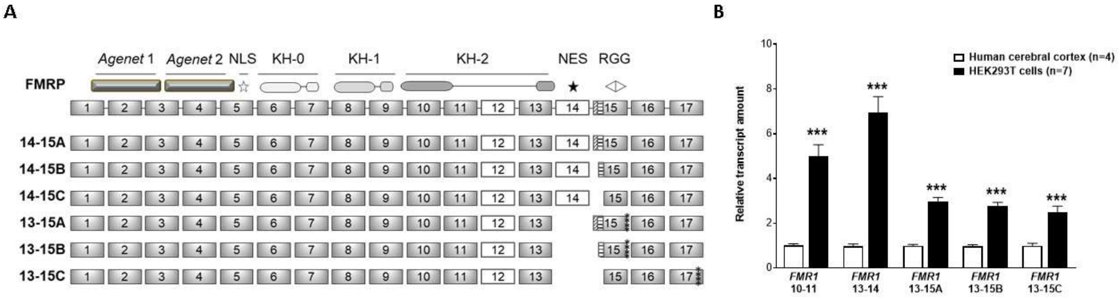

Supplementary Figure 4

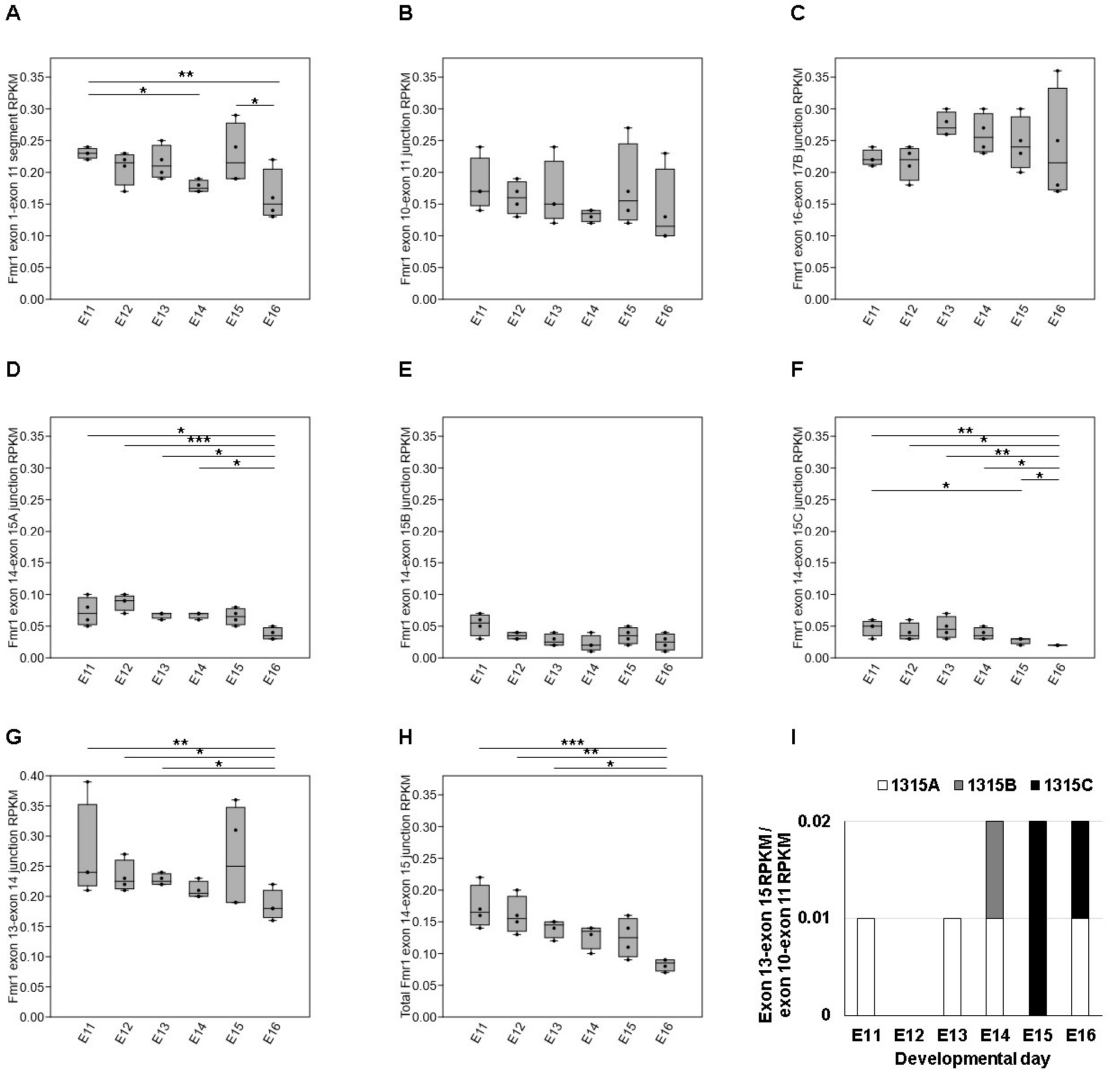

Supplementary Figure 5

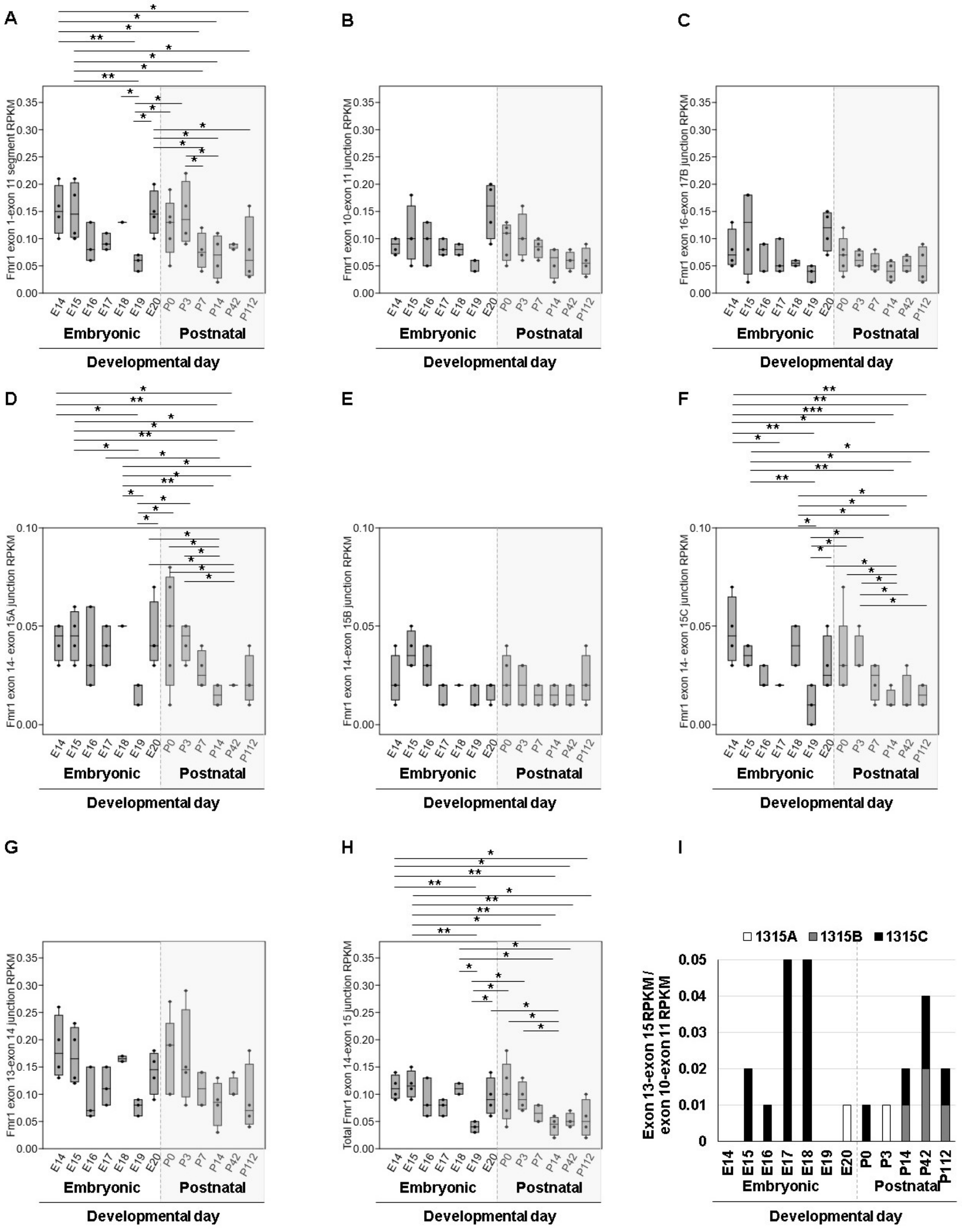

Supplement: Supplementary file 1 — Additional file 1. Supplementary human subjects and methods. Supplementary references. Oligonucleotide sequences employed in RT-qPCR and PCR for rat or human nucleic acid targets, or dsRNA for RNA interference. Accession numbers of sequence read archive (SRA) database project PRJEB26889 rat RNA-Seqs employed for Fmr1 BLAST. Significant Dunn´s post hoc test results for age pair comparisons of RPKM values obtained in BLAST searches addressing total Fmr1 or exon 14. Plot of RPKM values obtained by BLAST searches of Fmr1 sequences spanning junctions between exon 14 and usage of splice site 15A, 15B and 15C. Plot of RPKM ratio of exon 14/exon 15 splice site junction by the summation of all exon 14-exon 15 junctions. FMR1 mRNA RTqPCR of human aging cerebral cortex and non-transfected HEK293T cells. RPKM values quantifying Fmr1 sequences in the rat whole brain. RNA-Seq RPKM values quantifying Fmr1 sequences in the rat cerebellum. [file 12868_2022_711_MOESM1_ESM.pdf]
